# Quality and Maturity Detection of Korla Fragrant Pears via Integrating Hyperspectral Imaging with Multiscale CNN–LSTM

**DOI:** 10.3390/foods14203561

**Published:** 2025-10-19

**Authors:** Zhengbao Long, Tongzhao Wang, Zhijuan Zhang, Yuanyuan Liu

**Affiliations:** 1 College of Mechanical and Electrical Engineering, Tarim University, Alar 843300, China; 10757232267@stumail.taru.edu.cn (Z.L.); zzj120140006@taru.edu.cn (Z.Z.); 2 Agricultural Engineering Key Laboratory at Universities of Education Department of Xinjiang Uygur Autonomous Region, Tarim University, Alar 843300, China; 3 Xinjiang Production and Construction Corps (XPCC) Key Laboratory of Utilization and Equipment of Special Agricultural and Forestry Products in Southern Xinjiang, Alar 843300, China

**Keywords:** pears, firmness–soluble solids ratio, near-infrared hyperspectral imaging, convolutional neural network, Visualization

## Abstract

To address the limitations of single indices in comprehensively evaluating the quality of Korla fragrant pears, this study proposes the firmness–soluble solids ratio (FSR), defined as the ratio of average firmness (FI) to soluble solid content (SSC) for each individual fruit, as a novel index. Using 600 samples from five maturity stages with hyperspectral imaging (950–1650 nm), the dataset was split 4:1 by the SPXY algorithm. The findings demonstrated that FSR’s effectiveness in quantifying the dynamic relationship between FI and SSC during maturation. The developed multiscale convolutional neural network–long short-term memory (MSCNN–LSTM) model achieved high prediction accuracy with determination coefficients of 0.8934 (FI), 0.8731 (SSC), and 0.8610 (FSR), and root mean square errors of 0.9001 N, 0.7976%, and 0.1676, respectively. All residual prediction deviation values exceeded 2.5, confirming model robustness. The MSCNN–LSTM showed superior performance compared to other benchmark models. Furthermore, the integration of prediction models with visualization techniques successfully mapped the spatial distribution of quality indices. For maturity discrimination, hyperspectral-based partial least squares discriminant analysis and linear discriminant analysis models achieved perfect classification accuracy (100%) under five-fold cross-validation across all five maturity stages. This work provides both a theoretical basis and a technical framework for non-destructive evaluation of comprehensive quality and maturity in Korla fragrant pears.

## 1. Introduction

The Korla fragrant pear, belonging to *Pyrus bretschneideri*, is primarily cultivated in the northern margin of the Tarim Basin and the southern foothills of the Tianshan Mountains. This pear, renowned for its desirable quality traits such as sweet taste, thin skin, tender flesh, abundant juiciness, and rich aroma, occupies a key position in the fruit market [1,2]. However, its market competitiveness, postharvest storage longevity, and consumer acceptability are critically dependent on the precise regulation of fruit maturity and quality parameters [3]. Firmness (FI) and soluble solid content (SSC) are widely recognized as critical maturity indicators for Korla fragrant pears [4,5,6]. The maturity level, reflected by these parameters, directly influences sensory qualities such as taste and flavor, as well as postharvest storability and transport durability [7,8]. Therefore, accurately determining maturity is essential for ensuring the overall quality of fragrant pears. The evaluation of fruit maturity has been significantly advanced by studies utilizing key physical parameters like firmness and soluble solid content, as evidenced by foundational work on pears [9,10]. To complement and refine these established approaches, this study proposes the Firmness–Soluble Solids Ratio (FSR) as an integrated composite indicator [11]. This ratio aims to provide a more nuanced understanding of maturity dynamics. By quantifying the coordinated changes in SSC and FI, the FSR offers an enhanced theoretical framework for holistically assessing fruit quality and storage potential, thereby helping to reduce quality issues and economic losses from suboptimal harvest timing [12].

Currently, the quality assessment and maturity determination of Korla fragrant pears primarily rely on destructive physicochemical methods (e.g., FI measurement using texture analyzers and SSC determination using refractometers) and manual expertise [13,14]. These methods are inadequate to meet the growing demand for efficient, intelligent detection and postharvest decision-making in modern orchard management. Owing to its non-destructive nature, rapid operation, and unique capacity for spatial distribution visualization, near-infrared hyperspectral imaging (NIR-HSI) has gained increasing attention as a cutting-edge approach for internal quality evaluation in agricultural produce [15,16]. In contrast to conventional near-infrared spectroscopy, hyperspectral imaging enables simultaneous acquisition of spectral and spatial information, thereby facilitating visual mapping of quality-related attributes [17,18]. This technique has been effectively implemented for predicting individual quality indices such as SSC and FI in pears, as well as for discriminating maturity stages [5,13,19].

However, despite their numerous advantages, hyperspectral data are characterized by high dimensionality, considerable noise, and information redundancy, which hinder modeling. Although machine learning methods (e.g., partial least squares regression (PLSR), support vector machine (SVM), and principal component regression (PCR)) have been extensively employed [20,21,22], they have inherent limitations in processing complex data [23]. Linear models (e.g., PLSR and PCR) fail to decipher the underlying nonlinear relationships between spectral data and quality indexes. Nonlinear models (e.g., SVM) offer certain nonlinear processing capabilities, but their prediction performance is highly parameter sensitive and requires intricate optimization procedures. Additionally, these methods generally necessitate preprocessing or feature wavelength selection, while still facing challenges in attaining high-accuracy predictions for multiple key indices [24,25,26]. In this scenario, the rapid evolution of deep-learning technologies offers novel solutions to these issues [27,28]. For instance, convolutional neural networks (CNNs) can automatically extract deep-level spatial features from raw or preprocessed spectra, thereby reducing the reliance on manual feature engineering [29]. Conversely, long short-term memory networks (LSTMs) excel at capturing long-term dependencies in sequential data [30,31]. Although deep learning models (including CNNs, LSTMs, and their combinations) have demonstrated superior predictive capability in hyperspectral fruit quality assessment [32,33,34], existing approaches often fail to fully exploit multiscale spectral features and are rarely applied to the multi-task prediction of comprehensive indicators like FSR.

To address these limitations, this study develops a multiscale convolutional neural network combined with long short-term memory (MSCNN–LSTM) for predicting FI, SSC, and the proposed FSR index. The specific objectives of this study were: (1) to propose the FSR as a composite indicator for comprehensive maturity assessment of Korla fragrant pears; (2) to develop a MSCNN–LSTM model for accurate prediction of FI, SSC, and FSR; (3) to visualize the spatial distribution of these quality indices by integrating the model with hyperspectral imaging; and (4) to establish a maturity classification model for discriminating five distinct maturity stages.

## 2. Materials and Methods

### 2.1. Sample Preparation

Korla fragrant pears were harvested from an orchard in Alar City, Xinjiang Uygur Autonomous Region, China. To ensure sample representativeness, 50 trees aged 5–8 years were randomly selected across the orchard. At each of the five maturity stages (P1: 20 August 2024; P2: 31 August 2024; P3: 12 September 2024; P4: 24 September 2024; P5: 6 October 2024), 10 trees were randomly chosen for sampling. 12 fruits from each tree were collected from various canopy positions, including upper, middle, and lower layers in the four cardinal directions (east, south, west, and north).

The mean fruit weights across maturity stages were as follows: P1: 121.25 ± 7.63 g, P2: 126.63 ± 13.97 g, P3: 130.26 ± 10.94 g, P4: 142.35 ± 16.54 g, and P5: 144.45 ± 14.27 g (mean ± standard deviation). All fruits selected for analysis were free from mechanical damage, pest infestation, and disease, and exhibited uniform shape. After harvest, each fruit was individually protected with polyethylene foam net sleeves to minimize mechanical damage during transportation. All samples were transported to the laboratory and stored at 25 ± 0.5 °C and 80 ± 5% relative humidity for 24 h to equilibrate to room temperature. A total of 600 samples (120 per maturity stage) were finally cleaned, labeled, and prepared for subsequent analysis.

### 2.2. Hyperspectral Image Acquisition

Near-infrared hyperspectral images were acquired using a line-scanning imaging system (Jiangsu Shuanglihe Spectrum Technology Co., Ltd., Wuxi, China). The system comprised the following components: a hyperspectral camera covering a spectral range of 900–1700 nm with a resolution of 5 nm and 512 spectral bands; a motor-driven translation stage for sample presentation; a diffuse reflectance white reference panel for radiometric calibration; a computer equipped with SpecView software for data acquisition; two tungsten-halogen illumination sources.

**Figure 1:**
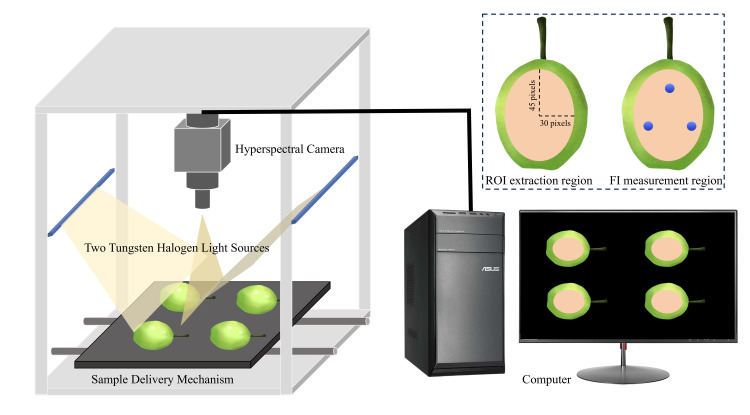
Data acquisition diagram.

The system was warmed for 30 min before acquisition to ensure stability. The hyperspectral image acquisition parameters for all samples were set as follows: the distance from the camera lens to the sample’s upper surface was 300 mm, the sample conveying speed was 0.8 cm/s, and the exposure time was 50 ms. During acquisition, the foam mesh was removed from the Korla fragrant pear and placed on the sample conveying mechanism, as illustrated in Figure 1.

### 2.3. Hyperspectral Image Correction and Spectral Data Extraction

Raw hyperspectral images are susceptible to camera dark current, noise, uneven illumination, and sample surface curvature. These factors prevent images from accurately reflecting the sample’s optical properties [8]. To eliminate such interference, this study employed the whiteboard-correction method to correct the original images. The correction was performed using Equation (1):

(1) $$R=\frac{R_{O}-R_{D}}{R_{W}-R_{D}}$$

where R is the corrected hyperspectral image; $R_{O}$ is the original hyperspectral image; $R_{W}$ is the white reference hyperspectral image captured by the standard calibration whiteboard; and $R_{D}$ is the black reference hyperspectral image captured when the camera lens is completely occluded.

After calibration, the region of interest (ROI) was defined on the surface of each fruit using ENVI 5.3 software to extract the spectral data. As illustrated in Figure 1, the ROI was positioned in the equatorial zone of the fruit, covering a consistent area for both imaging and subsequent destructive sampling. The ROI was defined as an elliptical region with its major axis aligned parallel to the fruit’s stem–calyx axis. The lengths of the long and short axes were set to 90 pixels and 60 pixels, respectively. Special attention was paid to maintaining this consistent orientation to avoid specular glare and ensure reproducible positioning. This region effectively excluded interference from the stalk and calyx, ensuring acquisition of representative spectral features from the fruit’s target region.

### 2.4. Determination of FSR in Korla Fragrant Pears

The formula for FSR is given in Equation (2) as follows:

(2) FSR=FI/SSC

where FSR represents the fruit Firmness–Soluble Solids Ratio; FI denotes the average FI, (N); SSC denotes the average SSC, (%).

Following hyperspectral image acquisition, fruit FI of Korla fragrant pears was determined by using a texture analyzer (GDTEST-01; Shimadzu Corporate Management Co., Ltd., Kyoto, Japan). A cylindrical puncture probe of diameter 5 mm was used for measurements following stress calibration. The measurement points were composed of three locations within the ROI depicted in Figure 1, with their specific positions illustrated in Figure 1. The texture analyzer parameters were configured as follows: downward speed, 0.50 mm/s; puncture speed, 0.50 mm/s; trigger force, 0.05 N; puncture depth, 10.00 mm; upward speed, 10.00 mm/s; displacement, 20.00 mm. The FI value for each fruit was calculated as the mean measurement calculated from the three points in the region shown in Figure 1.

SSC was determined using a digital refractometer (MDS-1; Shenzhen Meadows Biotechnology Co., Ltd., Shenzhen, China). Following firmness measurement, the peel within the predefined ROI was excised using a stainless-steel peeler, with the removal depth of 1.5–2.0 mm to ensure complete epidermis removal while minimizing flesh loss. All underlying pulp tissues (excluding the core) from this region were collected and homogenized mechanically to obtain juice for analysis. The refractometer was calibrated with ultrapure water prior to each measurement. A disposable dropper was used to transfer 2 mL of the homogenized juice onto the detection area, and the SSC values were recorded under a constant temperature environment (25 ± 0.5 °C). Triplicate determinations were then conducted for each fruit, with the mean value adopted as the SSC of the respective fruits.

### 2.5. Data Preprocessing and Partitioning

The spectral data were first refined by truncating the initial (900–950 nm) and terminal (1650–1700 nm) regions due to substantial interference from dark current and optical noise [35]. 421 contiguous spectral bands (950–1650 nm) were defined as effective spectral analysis range.

For model development and evaluation, the dataset was partitioned using the SPXY algorithm with a fixed random seed of 2. This partitioning was conducted in a stratified manner within each of the five maturity stages to prevent data leakage and to ensure a balanced representation of all maturity levels across both the calibration and prediction sets. This procedure resulted in a calibration set of 480 samples and a prediction set of 120 samples, corresponding to a 4:1 ratio. All machine learning and deep learning models, for both regression (FI, SSC, FSR) and classification tasks, were constructed and evaluated using this identical data partition, with model performance assessed based on the held-out prediction set.

### 2.6. Construction of Machine Learning Regression Models

To predict the FI, SSC, and FSR of Korla fragrant pears, three machine learning regression methods were established based on the hyperspectral data. PLSR was employed to extract latent variables maximizing the covariance between spectral and response variables, effectively handling multicollinearity. Support vector regression (SVR) with a radial basis function kernel (penalty coefficient of 90, kernel parameter of 1) was used to model nonlinear relationships. Additionally, PCR reduced data dimensionality using 10 principal components before constructing linear models to mitigate redundancy.

### 2.7. Construction of Deep-Learning Regression Models

#### 2.7.1. Residual Neural Network 18 (ResNet18)

The ResNet addresses the issues of gradient vanishing and degradation in deep networks via residual modules, enabling the network to be extended to a depth of 18 layers [36,37]. In this study, a one-dimensional CNN based on the ResNet18 architecture was constructed for high-dimensional feature extraction and regression prediction of the spectral data. The input layer of this residual architecture receives single-channel spectral sequences with a dimension of 1 × 421. The initial feature extraction stage employs a 7 × 1 convolutional kernel (Conv1d (1 → 64, stride 2, padding 3)), followed by batch normalization, a rectified linear unit (ReLU) activation function, and 3 × 1 max pooling (stride 2, padding 1) to achieve an initial feature dimensionality reduction and preserve edge information. The deeper feature extraction involves four residual groups, each containing two residual blocks, with the number of channels being 64, 128, 256, and 512 in sequence. Each residual block uses a 3 × 1 convolutional kernel, and the number of channels is adjusted via 1 × 1 convolutional skip connections to match the residual output dimensions, ensuring effective gradient propagation. Ultimately, features are compressed to 512 dimensions via a global average pooling layer, with prediction values output through a fully connected layer.

The training process employed the Adam optimizer with a learning rate of 0.0001, using mean square error ( MSE) as the loss function. The batch size was 32, and the number of iterations was 500; overfitting was suppressed by using an early stopping strategy. This architecture enhances the ability to capture deep spectral features through residual learning mechanisms, making it suitable for mining subtle patterns in high-dimensional spectral data and predicting the quality parameters of Korla fragrant pears.

#### 2.7.2. MSCNN

**Figure 2:**
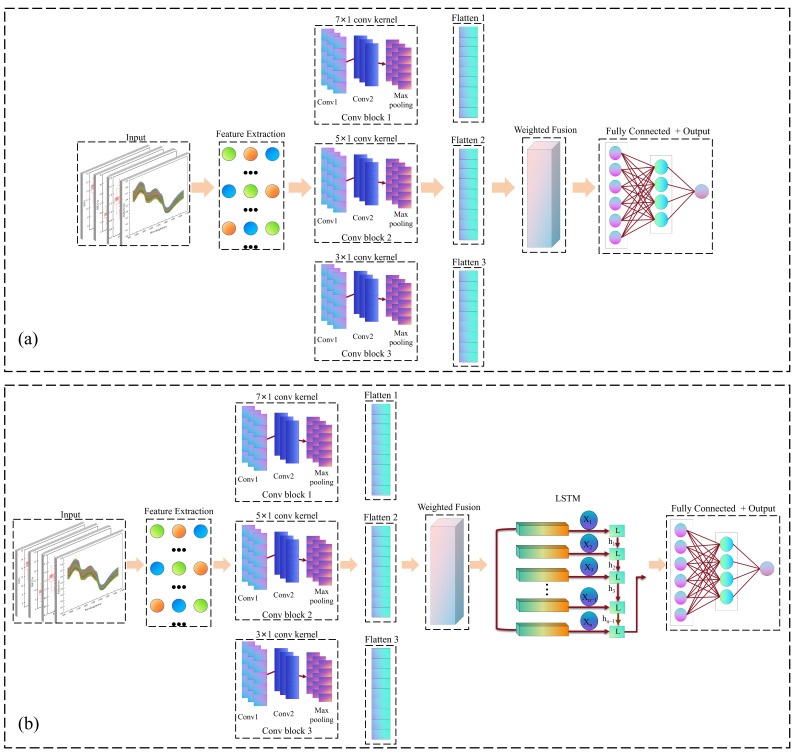
Deep-Learning model structure. (**a**) MSCNN, (**b**) MSCNN–LSTM.

CNN is a typical feed-forward neural network with a basic architecture composed of convolutional layers, pooling layers, activation functions, and fully connected layers [38]. However, its single-scale convolutional kernels have limitations in capturing features across different wavelength ranges in the spectral data. To address this concern, in this study, we designed a MSCNN that extracts multiscale features and achieves adaptive fusion through parallel branches (Figure 2a). Its core lies in using convolutional kernels of different sizes to capture features across local to global scales, thereby enhancing the model’s capacity to characterize complex spectral patterns. Multiscale feature extraction is achieved through three parallel branches: the small-scale branch uses two 3 × 1 convolutional layers (channel configuration 1 → 16 → 128) to capture local detailed features; the medium-scale branch employs two 5 × 1 convolutional layers (channel configuration 1 → 64 → 128) to extract medium-range features; the large-scale branch uses two 7 × 1 convolutional layers (channel configuration 1 → 128 → 128) to capture pattern information over a wider range. All convolutional layers have a stride of 2 and padding values of 1, 2, and 3, respectively, followed by batch normalization and ReLU activation functions. A 128 × 53 feature matrix (6784 dimensions after flattening) was formed after 2 × 1 max pooling.

In the feature-fusion stage, three learning weight vectors ($w_{1},w_{2},w_{3})$ were introduced to weight and fuse the features of the three branches, with dynamic weighted integration achieved via $F_{fusion}$. The fused features were dimensionally reduced via a fully connected layer (6784 → 128, with ReLU activation), and the final predicted values of Korla fragrant pear fruit quality were output through a linear layer, as shown in Equations (3)–(8):

(3) $$F_{fusion}=w_{1}\cdot F_{scale1}+w_{2}\cdot F_{scale2}+w_{3}\cdot F_{scale3}$$

(4) $$F_{scale1}\left(x\right)=MaxPool\left({Conv}_{3\times 1}^{2}\left(x\right)\right)$$

(5) $$F_{scale2}\left(x\right)=MaxPool\left({Conv}_{5\times 1}^{2}\left(x\right)\right)$$

(6) $$F_{scale3}\left(x\right)=MaxPool\left({Conv}_{7\times 1}^{2}\left(x\right)\right)$$

(7) $$H_{fusion}=ReLU\left({FC}_{128}\left(F_{fusion}\right)\right)$$

(8) $$y_{MSCNN}={FC}_{1}\left(H_{fusion}\right)$$

Training was performed using the Adam optimizer with a learning rate of 0.0001, MSE as the loss function, a batch size of 32, and 1000 iterations. Through the complementary fusion of multiscale features, this architecture helped enhance the comprehensive capture capability of subtle spectral features and global trends, making it suitable for predicting the quality parameters of Korla fragrant pears.

#### 2.7.3. MSCNN–LSTM

A LSTM unit dynamically filters temporal information through the cooperative regulation of input gates, forgetting gates, and output gates [39,40]. This effectively addresses the gradient vanishing problem of the conventional recurrent neural networks and enables the unit to capture long-term dependencies in the sequence data [38,41]. In this study, we proposed the MSCNN–LSTM model that combines the multiscale feature-extraction capability of MSCNN with the time-series modeling advantage of LSTM to uncover the potential correlations between spectral bands, as illustrated in Figure 2b. The model is an extension of MSCNN. In the feature extraction stage, it reuses the multiscale parallel branches of MSCNN, producing 128-dimensional fusion features $H_{fusion}$ after pooling, dimension alignment, and weighted fusion. To adapt to the sequence input format of LSTM, $H_{fusion}$ is reshaped into temporal input via dimension expansion (with the time step of 1) and fed into a single-layer LSTM with an input dimension of 128 and a hidden dimension of 128. The hidden state $H_{lstm}$ from the final time step is regarded as the temporal feature representation, whereas the final prediction is produced through a linear layer, as shown in Equations (9) and (10):

(9) $$H_{lstm}=LSTM\left(H_{fusion}\right)$$

(10) $$y_{MSCNN-LSTM}={FC}_{1}\left(H_{lstm}\right)$$

### 2.8. Classification Models

Three classification algorithms were implemented for maturity stage discrimination: Partial Least Squares-Discriminant Analysis (PLS-DA), SVM with a radial basis function kernel, and Linear Discriminant Analysis (LDA). The PLS-DA model projected spectral data into a latent space to maximize separation between maturity categories [42], while the SVM classifier was configured with a penalty parameter C of 10 and gamma set to “scale” [43,44]. For the LDA classifier, recursive feature elimination was tightly coupled with the model training process to enhance discriminative performance through feature selection [16,43].

### 2.9. Model Evaluation

#### 2.9.1. Regression Model Evaluation

The regression models were evaluated using the coefficient of determination ($R^{2}$), the root mean square error (RMSE), and the residual prediction deviation (RPD). $R^{2}$ quantifies the proportion of variance explained by the model, with values approaching 1 indicating a superior fit. RMSE reflects the average deviation between predicted and actual values. RPD evaluates the model’s robustness and generalization capability; a model is generally considered to have excellent predictive power when RPD exceeds 2.5 [45,46].

#### 2.9.2. Classification Model Evaluation

Maturity classification model performance was evaluated using a rigorous five-fold cross-validation framework. To prevent data leakage and ensure a robust performance estimate, the complete modeling pipeline—including all preprocessing steps and the RFE feature selection for LDA—was executed independently within each fold. Specifically, operations such as feature scaling and feature selection were calibrated exclusively on the training portion of each fold before being applied to the corresponding test set. The evaluation metrics comprising accuracy, precision, recall, and F1-score were calculated from the aggregated predictions across all cross-validation folds, providing an unbiased assessment of the models’ generalization capability [47]. All models were evaluated using this identical, stringent cross-validation protocol.

## 3. Results and Analysis

### 3.1. Spectral Analysis

**Figure 3:**
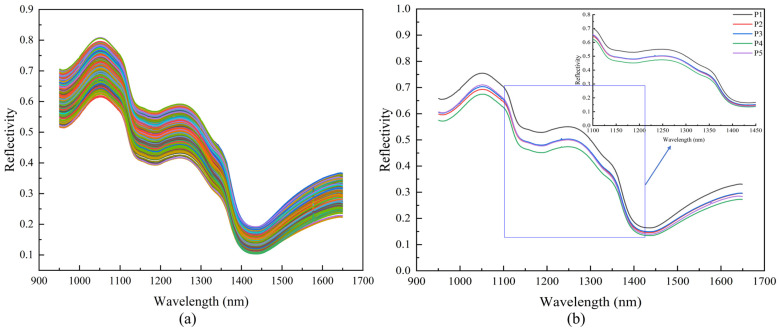
Reflectance spectral curves. (**a**) Raw, (**b**) Average curves of different maturities.

The 600 hyperspectral datasets of Korla fragrant pears acquired in this study covered the 950–1650 nm wavelength range. Although the original spectra of pears at different maturity stages (Figure 3a) displayed individual variability, the reflectance showed regular, fluctuating patterns. The average spectrum (Figure 3b) clearly highlighted the differentiation of absorption characteristics across various maturity stages. This interval encompassed spectral responses related to fruit moisture and carbohydrates, with reflectance differences resulting from the stretching vibrations of O–H and C–H bonds.

Absorption peaks in the reflectance spectra of Korla fragrant pears corresponded to specific absorption bands: 900–1000 nm, 1100–1200 nm, and 1400–1500 nm corresponded to the second overtone absorption region of O–H bonds, the second overtone absorption region of C–H bonds, and the combination band absorption region of O–H bonds, respectively [48,49,50]. Moreover, the absorption peak at 970 nm was associated with O–H bonds [51,52], the weak absorption peak near 1080 nm was related to the third harmonic of N–H bonds [53], and the absorption band at 1270 nm was linked to the second overtone of C–H stretching vibrations in -CH/-CH_2_ groups [54]. The flat absorption peak at 1200 nm within the 1150–1250 nm range corresponded to the C–H overtone region of soluble sugars [55], directly reflecting SSC dynamics. The differences in the absorption valley near 1450 nm during the ripening stages reflected the changes in fruit moisture status with maturity [56].

### 3.2. Variation Patterns of FI, SSC, and FSR with Maturity

#### 3.2.1. Variation Patterns of FI

**Figure 4:**
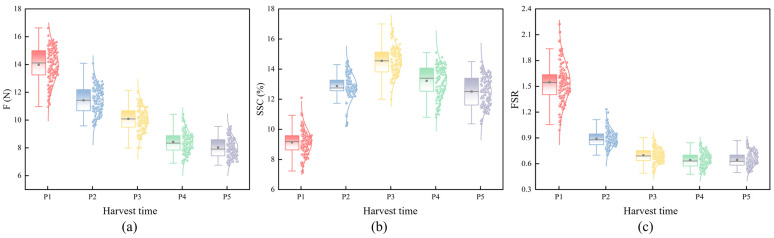
Variations in quality indices with maturity. (**a**) FI, (**b**) SSC, (**c**) FSR.

As illustrated in and Figure 4a, the FI of Korla fragrant pears decreased continuously with advancing maturity, with a 42.5% reduction from P1 (13.98 ± 1.24 N) to P5 (8.03 ± 0.75 N). A high variability in FI was noted at the P1 stage (range: 10.97–16.63), signifying differences in the accumulation of cell wall structural components (e.g., cellulose and pectin) owing to environmental variations (e.g., light and nutrient availability) during fruit development. During the P2–P5 stages, FI declined uniformly; the boxplot in Figure 4a flattened with a decreasing standard deviation (from 1.24 N to 0.75 N). This observation reflected the systematic disintegration of the cell wall, driven by increased pectinase and cellulase activities, ultimately leading to texture softening.

#### 3.2.2. Variation Patterns of SSC

The SSC of Korla fragrant pears demonstrated a unimodal variation trend of “accumulation–consumption” with maturity, as illustrated in Table 1 and Figure 4b. P1–P3 represented the rapid SSC accumulation stage, during which SSC escalated from 9.13 ± 0.84% to 14.55 ± 0.93%, with a 59.4% surge. The upward shift in the boxplot was accompanied by the highest variability (standard deviation: 0.93%), which can be attributed to individual variation in sugar synthesis at P3. These differences were associated with environmental heterogeneity in light intensity and nutrient supply. P4–P5 marked the consumption and decline stage, with SSC dropping to 13.22 ± 1.02% (P4) and 12.51 ± 0.99% (P5). The boxplot shifted downward, indicating a decrease in variability. This observation could be attributed to enhanced respiratory metabolism during the overripe stage, with the rate of sugar consumption exceeding the rate of synthesis, resulting in a reduction in the net accumulation of soluble sugars.

#### 3.2.3. Variation Patterns of FSR

The dynamic changes in FSR were categorized into two stages, as depicted in Table 1 and Figure 4c. P1 was the texture-dominant stage, with an average FSR of 1.55 ± 0.22. The discordant state was characterized by high FI and low SSC in the fruits at this stage, which manifested as a hard and crisp texture but insufficient sugar content, leading to poor flavor. P2–P5 constituted the sugar-dominant stage at which FSR fell continuously from 0.89 ± 0.10 (P2) to 0.64 ± 0.08 (P4) (a 28.1% reduction), followed by a slight increase to 0.65 (P5). Meanwhile, the boxplot in Figure 4c gradually decreased in height, indicating a decrease in data variability. This process was primarily driven by pectinase-mediated reduction in FI. Starch hydrolysis and photosynthate translocation promoted SSC accumulation from P2 to P3. Respiratory metabolism caused SSC to decline from P4 to P5, and the rate of FI attenuation slowed down, ultimately resulting in a coordinated state of softened texture and moderate sugar content.

The dynamic variation pattern of the FSR is pivotal for evaluating the maturity of Korla fragrant pears and informing harvest decisions. Unlike single indices such as FI or SSC, the FSR effectively integrates multiple quality aspects. This integration provides a theoretical framework for informing harvest decisions, pending future agronomic validation. Based on the FSR patterns observed in this study, P4 (FSR of 0.64 ± 0.08) may represent a potential window for fresh consumption, as it corresponds to a softened texture (FI of 8.43 ± 0.76 N) while maintaining a high sugar level (SSC of 13.22 ± 1.02%). Conversely, the P2 stage (FSR of 0.89 ± 0.10), characterized by a higher FSR and substantial sugar content (SSC of 12.87 ± 0.82%), could be more suitable for long-term storage due to better-preserved cellular structure. The harvesting timing can be potentially matched to diverse consumer demands by analyzing the stage-specific characteristics of FSR and its intrinsic association with fruit physiological metabolism.

### 3.3. Analysis of Regression Models

#### 3.3.1. Analysis of Machine Learning Regression Models

**Figure 5:**
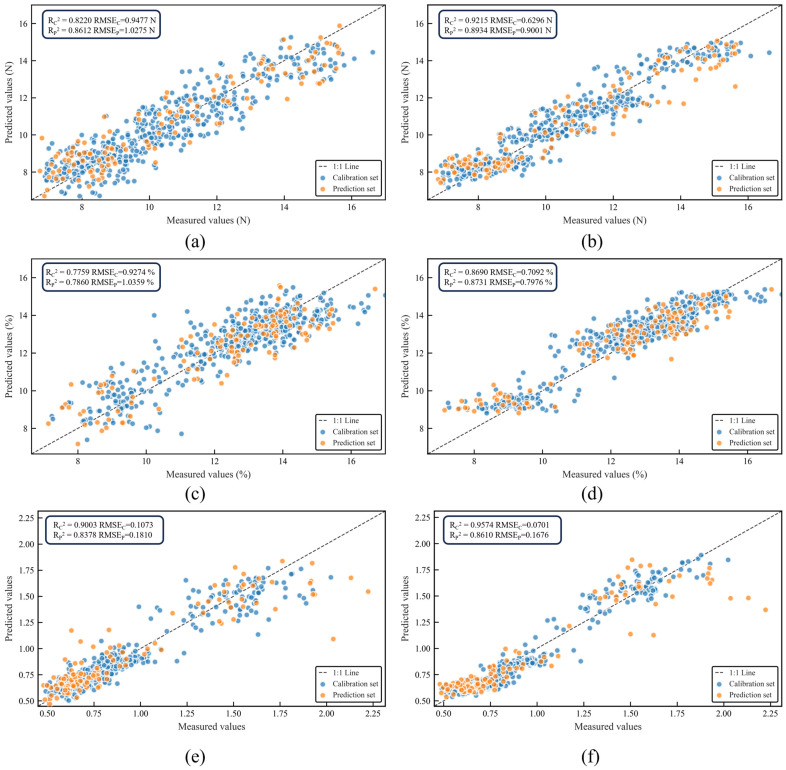
Scatterplots of optimal prediction models. (**a**) PCR model for FI, (**b**) MSCNN–LSTM model for FI, (**c**) PCR model for SSC, (**d**) MSCNN–LSTM model for SSC, (**e**) SVR model for FSR, (**f**) MSCNN–LSTM model for FSR.

The prediction results of the machine learning models are shown in Table 2. In predicting FI, PCR performed optimally, with the $R_{P}^{2}$ of 0.8612, ${RMSE}_{P}$ of 1.0275 N, and RPD of 2.6837 (Figure 5a). Nonetheless, its predicted values for samples with high FI at the P1 stage were lower than the actual values (FI > 14 N). In predicting SSC, although PCR was the optimal models, with the $R_{P}^{2}$ of 0.7860 and ${RMSE}_{P}$ of 1.0359%, its RPD was 2.1616, which did not meet the reliability threshold of 2.5. This model was suitable only for rough screening (Figure 5c). In comparison, SVR for SSC, with the $R_{C}^{2}$ of 0.8009, $R_{P}^{2}$ of 0.7189, and a decline of 10.2%, exhibited the risk of overfitting. In predicting FSR, SVR performed optimally owing to its nonlinear kernel function, with the $R_{P}^{2}$ of 0.8378, ${RMSE}_{P}$ of 0.1810, and RPD of 2.4829. Nevertheless, the prediction variability increased with a high FSR (FSR > 1.5 at the P1 stage) (Figure 5e), indicating the model’s insufficient capability to characterize discordant quality traits (high FI and low SSC). None of the three models achieved high-precision prediction of multiple indices. These findings highlight the inherent limitations of machine learning models in capturing nonlinear relationships between spectral data and quality in this study.

#### 3.3.2. Analysis of Deep-Learning Regression Models

This study constructed ResNet18, MSCNN, and MSCNN–LSTM models to address the limitations of machine learning models in modeling high-dimensional, nonlinear spectral data. Their predictive performances are listed in Table 2.

ResNet18 demonstrated a strong feature-fitting capability due to its deep residual structure; however, it exhibited a high risk of overfitting. Numerical evidence beyond the $R^{2}$ discrepancy includes the exceptionally low training loss (near zero) and substantially higher validation loss observed during model training. For example, it achieved the $R_{c}^{2}$ of 0.9949 and $R_{P}^{2}$ of 0.8591 for SSC, representing a 13.58% performance decrease, while the training ${RMSE}_{C}$ was only 0.1400% compared to the prediction ${RMSE}_{P}$ of 0.8404%. This significant gap between training and prediction errors provides additional proof of overfitting. MSCNN utilized 3 × 1, 5 × 1, and 7 × 1 multiscale convolutional kernel to parse multirange spectral features, outperforming machine learning models in predicting FI ( $R_{P}^{2}$ of 0.8506) and SSC ( $R_{P}^{2}$ of 0.8581); however, its performance in predicting FSR ( $R_{P}^{2}$ of 0.8357) was inferior to that of SVR.

The MSCNN–LSTM architecture not only preserved multiscale spectral details but also explored potential temporal correlations between spectral bands. In predicting FI, it achieved the highest $R_{P}^{2}$ of 0.8934, the lowest ${RMSE}_{P}$ of 0.9001 N, and RPD of 3.0634. The $R_{P}^{2}$ values were 2.55% and 5.03% higher than those of ResNet18 and MSCNN, respectively. The MSCNN–LSTM model of FI overcame the inadequacy of machine learning models in predicting samples with high FI at the P1 stage (Figure 5b). In predicting SSC, the $R_{P}^{2}$ was 0.8731, which was 1.63% and 1.75% higher than those of ResNet18 and MSCNN, respectively. Especially, it alleviated the underestimation bias of machine learning models for samples with high SSC (>15%) and demonstrated stronger generalization and stability, with the RPD of 2.8076 (Figure 5d). In predicting FSR, its $R_{P}^{2}$ was 0.8610, which was 0.82% and 3.03% higher than those of ResNet18 and MSCNN, respectively. The ${RMSE}_{P}$ decreased to 0.1676 and RPD reached 2.6825, exceeding the 2.5 threshold. The prediction capability was optimized for samples with a high FSR at the P1 stage (Figure 5f).

The optimal machine learning models in this study were limited by index dependence (e.g., PCR for FI, PCR for SSC, and SVR for FSR), with poor performance in predicting extreme values, and none achieved RPD of >2.5 simultaneously. Of the various deep-learning models, ResNet18 failed to achieve optimal results due to overfitting, whereas MSCNN was limited by its lack of temporal modeling capabilities. In contrast, the MSCNN–LSTM achieved the RPD of >2.5 and enhanced model stability for all three indices (FI, SSC, and FSR) by synergistically mining the spatial and temporal features.

### 3.4. Visualization and Analysis of Quality Indexes for Korla Fragrant Pears

**Figure 6:**
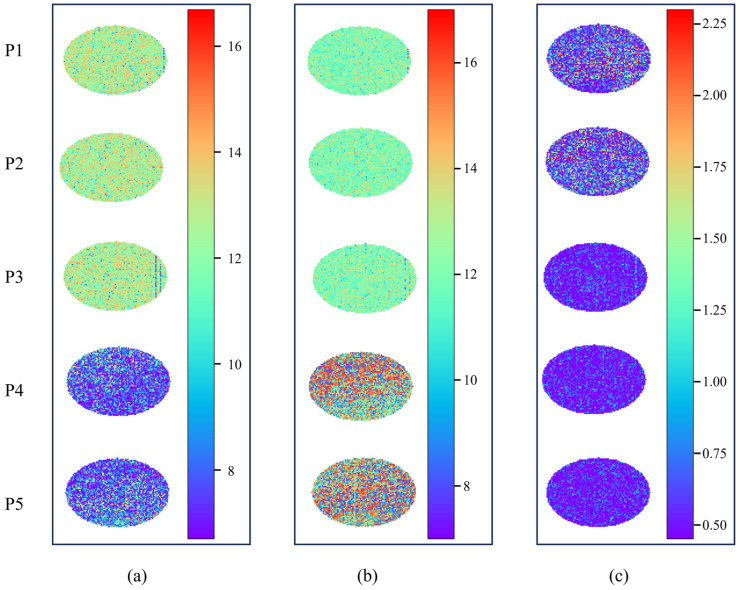
Visualization results of five maturities for three quality indices (**a**) FI, (**b**) SSC, (**c**) FSR.

Hyperspectral imaging enables the simultaneous acquisition of spectral information and spatial distribution traits of fruits [57]. In this study, the optimal MSCNN–LSTM models were applied to generate prediction maps by processing the spectrum of each individual pixel across the fruit surface. For visualization, a rectangular region measuring 90 × 60 pixels (major × minor axes) was randomly selected on the fruit surface. The dynamic evolution of quality indexes with maturity and the spatial heterogeneity of fruit quality were intuitively visualized using color gradients. The spatial distributions of fruit FI, SSC, and FSR are presented in Figure 6.

Specifically, distinct stage-specific features were evident for the three quality indexes during the ripening process from P1 to P5. Images of the P1–P3 stages contained extensive red and orange regions, as depicted in Figure 6a, which corresponded to high FI values, establishing the integrity of fruit cell wall structures. In the P4–P5 stages, the imagery gradually transitioned to the dominance of cyan and blue, with FI values decreasing to <10 N. This observation reflected the reduction in firmness induced by cell wall degradation during ripening, consistent with the trend of mean FI values presented in Table 1. In contrast, blue and cyan regions predominated at P1–P3 in Figure 6b, corresponding to lower SSC values. The orange and red regions gradually expanded, resulting in increased SSC, which was driven by progressive sugar accumulation via photosynthate translocation to the fruit at P4–P5. For the FSR visualization (Figure 6c), the color gradient of FSR in the P1 and P2 stages spanned multiple hues owing to the inverse variation between FI and SSC, which indicated a discordant state characterized by high firmness and low sugar content. After P3, the concurrent decrease in FI and increase in SSC led to a gradual reduction in FSR. This trend was visually dominated by blue in the spatial maps, signifying enhanced coordination between texture and sugar content, accompanied by a progressive decline in spatial heterogeneity.

Hyperspectral pixel-wise visualization overcomes the spatial constraints of point-based detection methods, enabling the accurate mapping of microscopic distribution variations in quality indexes. This method provides intuitive spatial support for dissecting the FI–SSC–FSR synergistic relationship and optimizing maturity grading, thereby validating the application potential of the MSCNN–LSTM model in complex quality characterization.

### 3.5. Analysis of Maturity Prediction Models

**Figure 7:**
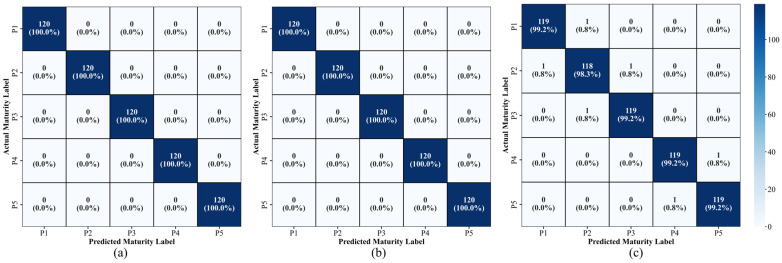
Optimal confusion matrices of the test set. (**a**) LDA, (**b**) PLS-DA, (**c**) SVM.

The classification performance of Korla fragrant pears for five maturity stages (P1–P5) is presented in Table 3 and Figure 7. The accuracy, precision, recall, and F1-score of PLS-DA and LDA models were all 100% in both training and test sets (Figure 7a,b). These results indicated perfect maturity classification performance, which reflected the strong linear separability between the hyperspectral data and harvest maturity. Consequently, both models effectively captured the continuous spectral variations occurring during fruit maturation. This performance can be attributed to the capacity of PLS-DA to simultaneously extract spectral features and maturity-related information through latent variable decomposition. The method suppressed high-dimensional noise while maximizing inter-class separation, thereby enhancing discriminative ability even for samples with low signal-to-noise ratios. In contrast, LDA projected the high-dimensional data into an optimal discriminant subspace by maximizing the ratio of inter-class to intra-class scatter, thus improving the accurate separation of boundary samples.

In comparison, the generalization capability of the SVM model was slightly inferior to that of PLS-DA and LDA (test set accuracy: 99.00% ± 0.62%). The misclassifications were concentrated in adjacent maturity stages (P1 vs. P2, P2 vs. P3, and P4 vs. P5), as shown in Figure 7c, with a misclassification rate of 0.8% (1 out of 120 samples) for each adjacent stage, and no cross-stage misclassifications. This phenomenon could be explained by the continuous physiological changes (e.g., chlorophyll degradation and sugar accumulation) that occur during fruit maturation, resulting in higher spectral feature overlap between adjacent stages than between nonadjacent stages, thereby exacerbating the difficulty in classifying boundary samples. SVM could enhance nonlinear fitting capability via kernel functions, but it was more sensitive to the distribution of training data and amplified subtle spectral overlap effects. In contrast, the linear discriminant mechanisms of LDA and PLS-DA were well-suited for the spectral patterns associated with the maturation process.

## 4. Discussion

While machine learning models are widely used in fruit quality detection, their predictive accuracy remains limited, as illustrated in Table 4. In contrast, the proposed MSCNN–LSTM model enables the automatic optimization of feature extraction via an end-to-end learning paradigm, facilitating the mining of deep-seated patterns from raw spectral data. Consequently, superior predictive performance was obtained for key quality indexes of Korla fragrant pears: the $R_{P}^{2}$ of FI reached 0.8934 (the ${RMSE}_{P}$ was 0.9001 N), $R_{P}^{2}$ of SSC attained 0.8731 ( ${RMSE}_{P}$ was 0.7976%), and $R_{P}^{2}$ of FSR touched 0.8610 ( ${RMSE}_{P}$ was 0.1676). These results underscore the advantages of deep learning in modeling complex hyperspectral relationships, combining MSCNN’s capacity to capture multi-scale spectral features and LSTM’s ability to model sequential spectral correlations, thereby improving information extraction across wavelengths [38].

Deep learning, particularly the integration of CNN and HSI, has shown strong potential in food quality analysis. For example, [32] used CNN to predict peach FI and SSC with $R_{P}^{2}$ values of 0.8523 and 0.8774, respectively. Reference [58] reported the $R_{P}^{2}$ of 0.8370 for SSC in mandarins, while [13] achieved the $R_{P}^{2}$ of 0.5120 for crown pears SSC. The predictive accuracy of the current model for FI ( $R_{P}^{2}$ was 0.8934) and SSC ( $R_{P}^{2}$ was 0.8731) compares favorably with these previous studies. Although direct comparative data for FSR are lacking, the high $R_{P}^{2}$ (0.8610) confirms the model’s effectiveness in quantifying the dynamic synergistic association between FI and SSC, offering a novel quantitative tool for comprehensive fruit maturity and quality assessment.

It is important to note that direct cross-study comparisons require caution, as variables such as species-specific traits, spectral range, and sample size can considerably influence model performance. For instance, Table 4 reveals inherent disparities in compositional complexity and spectral response characteristics across diverse fruits, including pears, peaches, and apricots. Additionally, deep learning may face challenges such as overfitting in low-complexity spectral data and high computational costs [59]. Therefore, model selection should align with data characteristics and application constraints [60,61]. In this study, overfitting was mitigated through early stopping and structural optimization (e.g., multi-scale kernels). Future work should validate model generalizability across regions and seasons, improve robustness through expanded datasets and transfer learning, and explore lightweight deployments for practical use.

In summary, the MSCNN–LSTM model overcomes the limitations of machine learning models by synergistically mining spatial and temporal features. The proposed FSR index enriches the dimensionality of comprehensive fruit quality evaluation, and the integrative approach provides valuable theoretical and technical insights for maturity assessment and quality control.

**Table 4:** Model comparison.

Index	Fruit Type	Spectral Range	Model	$R_{P}^{2}$	${RMSE}_{P}$	Reference
SSC	Pear	950–2500 nm	MSC-CARS-PLS	0.8284	0.3655	[62]
	Apricot	180–1100 nm	SG-MLP-XGBoost	0.7182	1.7400	[63]
	Pear	380–1030 nm	RAW-PLSR	0.8320	0.3300	[8]
FI	Peach	400–1000 nm	Nor-RF-MLR	0.8200	1.0270	[64]
	Apple	200–1100 nm	RAW-Ridge	0.8552	0.3386	[65]
	Pear	350–1100 nm	RAW-PLSR	0.8100	3.8100	[50]

Note: MSC, Multiplicative Scatter Correction; CARS, Competitive Adaptive Reweighted Sampling; PLS, Partial Least Squares; SG, Savitzky–Golay Smoothing; MLP, Multilayer Perceptron; XGBoost, Extreme Gradient Boosting Machine; RAW, Raw Data; Nor, Normalization; RF, Random Forest; MLR, Multiple Linear Regression; Ridge, Ridge Regression.

## 5. Conclusions

This study developed a framework for assessing Korla fragrant pear quality using NIR-HSI and deep learning. The proposed FSR parameter captured the relationship between FI and SSC, offering an alternative to single-index evaluations. The MSCNN-LSTM model showed a comparative advantage over conventional machine learning and single-structure deep learning models in predicting FI, SSC, and FSR. Additionally, the integration of NIR-HSI visualization mapped the spatial distribution of these quality indices, illustrating heterogeneity within fruits. Classification models confirmed that the maturity stages were linearly separable under the experimental conditions. These findings are specific to Korla fragrant pears in this study, and their applicability to other varieties or commercial settings requires further investigation.

## Figures and Tables

**Table 1:** Statistical results of FI, SSC, and FSR at different maturity stages.

Indicator	Statistic	P1	P2	P3	P4	P5
FI (N)	Minimum	10.97	9.58	7.99	6.90	6.75
	Maximum	16.63	14.09	12.13	10.42	9.55
	Mean	13.98	11.42	10.09	8.43	8.03
	Standard deviation	1.24	0.99	0.88	0.76	0.75
SSC (%)	Minimum	7.13	10.23	12.00	10.80	10.37
	Maximum	12.10	14.53	17.00	15.10	14.50
	Mean	9.13	12.87	14.55	13.22	12.51
	Standard deviation	0.84	0.82	0.93	1.02	0.99
FSR	Minimum	0.99	0.70	0.49	0.48	0.50
	Maximum	2.22	1.23	0.90	0.84	0.87
	Mean	1.55	0.89	0.70	0.64	0.65
	Standard deviation	0.22	0.10	0.09	0.08	0.08

**Table 2:** Results of model construction.

Index	Model	Calibration Set		Prediction Set		
		$R_{c}^{2}$	${RMSE}_{C}$	$R_{P}^{2}$	${RMSE}_{P}$	***PRD***
FI (N)	SVR	0.7740	1.0678	0.7853	1.2777	2.1581
	PLSR	0.7768	1.0612	0.8355	1.1183	2.4658
	PCR	0.8220	0.9477	0.8612	1.0275	2.6837
	MSCNN	0.8869	0.7553	0.8506	1.0657	2.5873
	Resnet18	0.9922	0.1987	0.8712	0.9895	2.7867
	MSCNN–LSTM	0.9215	0.6296	0.8934	0.9001	3.0634
SSC (%)	SVR	0.8009	0.8743	0.7189	1.1853	1.8893
	PLSR	0.7279	1.0221	0.7275	1.1689	1.9158
	PCR	0.7759	0.9274	0.7860	1.0359	2.1616
	MSCNN	0.8815	0.6744	0.8581	0.8437	2.6542
	Resnet18	0.9949	0.1400	0.8591	0.8404	2.6645
	MSCNN–LSTM	0.8690	0.7092	0.8731	0.7976	2.8076
FSR	SVR	0.9003	0.1073	0.8378	0.1810	2.4829
	PLSR	0.8254	0.1419	0.7949	0.2035	2.2082
	PCR	0.8495	0.1318	0.8317	0.1844	2.4374
	MSCNN	0.8996	0.1076	0.8357	0.1822	2.4671
	Resnet18	0.9859	0.0403	0.8539	0.1718	2.6159
	MSCNN–LSTM	0.9574	0.0701	0.8610	0.1676	2.6825

**Table 3:** Performance of maturity classification models.

Model	Training Set				Test Set			
	Accuracy (%)	Precision (%)	Recall (%)	F1-Score (%)	Accuracy (%)	Precision (%)	Recall (%)	F1-Score (%)
LDA	100.00	100.00	100.00	100.00	100.00	100.00	100.00	100.00
PLS-DA	100.00	100.00	100.00	100.00	100.00	100.00	100.00	100.00
SVM	99.75 ± 0.08	99.75 ± 0.08	99.75 ± 0.08	99.75 ± 0.08	99.00 ± 0.62	99.05 ± 0.59	99.00 ± 0.62	99.00 ± 0.62

## Data Availability

The original contributions presented in the study are included in the article. Further inquiries can be directed to the corresponding authors.

## Abbreviations

The following abbreviations are used in this manuscript:

FSR	Firmness–soluble solids ratio
FI	Firmness
SSC	Soluble solid content
MSCNN–LSTM	Multiscale convolutional neural network–long short-term memory
SVM	Support vector machine
PCR	Principal component regression
NIR-HSI	Near-infrared hyperspectral imaging
ROI	Region of interest
R^2^	Coefficient of determination
RMSE	Root mean square error
RPD	Residual prediction deviation
SVR	Support vector regression
PLS-DA	Partial least squares-discriminant analysis
LDA	Linear discriminant analysis
